# The prevalence, clinical reasoning and impact of non-standard anti-tuberculosis regimens at the initial prescription

**DOI:** 10.1038/s41598-024-55273-5

**Published:** 2024-03-07

**Authors:** Rou-Tsern Chen, Chih-Yu Liu, Shu-Yung Lin, Chin-Chung Shu, Wang-Huei Sheng

**Affiliations:** 1 Department of Nursing, National Taiwan University Hospital, Taipei, Taiwan; 2 College of Medicine, National Taiwan University, Taipei, Taiwan; 3 Department of Internal Medicine, National Taiwan University Hospital, No 7, Chung Shan South Road, Taipei, Taiwan

**Keywords:** Drug susceptibility, Factors, Non-standard regimen, Tuberculosis, Diseases, Medical research, Risk factors, Signs and symptoms

## Abstract

Regarding clinically-concerning non-standard initial anti-tuberculous (TB) regimens, few studies have examined their prevalence, risk factors and impacts. We recruited patients with drug susceptible TB and non-standard initial anti-TB regimens (NSTB group) and matched them with patients with standard initial regimens (STB group) in a 1:1 ratio. The risk factors and outcomes were analyzed. During the 11-year study period, we analyzed 50 (3.7%) patients with NSTB from a total set of 1337 patients with drug-susceptible TB. Pyrazinamide (60%) was the drug most commonly not prescribed in the NSTB group, followed by ethambutol (34%). Multivariable logistic regression identified independent risk factors as underlying eye disease (adjusted odds ratio [aOR]: 8.869; 95% CI 2.542–30.949; p = 0.001), gout/hyperuricemia (aOR: 4.012 [1.196–13.425]; p = 0.024), and liver disease (aOR: 12.790 [3.981–41.089]; p < 0.001). The NSTB group had longer treatment durations (281 ± 121 vs. 223 ± 63 days; p = 0.003) and more occurrences of treatment interruption (26% vs. 8%; p = 0.021) than the STB group. In conclusion, NSTB occurs in around 3.7% of patients and is associated with longer treatment and more treatment interruption. The risk factors might include underlying liver and eye diseases, and gout. Further studies to improve non-standard initial regimens and prevent negative outcomes are warranted.

## Introduction

Tuberculosis (TB) is still an important infectious disease and remains a leading cause of mortality worldwide^1^. In 2020, an estimated 5.8 million people had active TB, and 1.3 million TB related deaths were recorded^2^. Early detection and good treatment are important components of the End TB Strategy, as they may decrease TB transmission and control the pulmonary complications as well as mortality^3^.

The standard TB treatment for drug susceptible pulmonary TB includes an initial intensive phase using four anti-TB medications (isoniazid, rifampin or rifabutin, ethambutol and pyrazinamide) for two months, and this approach has shown favorable outcomes at the end of treatment in over 92% of cases in clinical trials^4,5^. The compliance rate for prescribing the standard anti-TB treatment at commencement is not 100%, and the actual rate remains unclear. The clinical reasoning for prescribing a non-standard initial anti-TB regimen might involve the fragility of elderly patients, underlying comorbidities or abnormal laboratory data. However, little clinical analysis has been focused on the reasons.

Notably, because non-standard initial TB treatment might be an obstacle for treatment completion due to the prolonged treatment course^6^ and may lead to TB recurrence^7^, the occurrence percentage and the risk factors warrant study if further improvements are to be made. Therefore, we conducted this retrospective study to investigate the real-world occurrence rate of non-standard initial TB treatment, its risk factors, and its clinical impact.

## Methods

### Patient recruitment

We reviewed the mycobacterial cultures of respiratory specimens positive for *Mycobacterium tuberculosis* and its drug susceptibility tests as well as medical records in the study hospital’s tuberculosis registry and electronic record system. Patients who were aged ≥ 20 years, had a diagnosis of first-line drug-susceptible pulmonary tuberculosis (TB) and received anti-TB drugs from January 2010 to December 2020 were reviewed. In this study, we did not enroll isolated extra pulmonary tuberculosis. We reviewed the prescriptions of the initial anti-TB regimens and selected the patients who did not initially receive the standard intensive regimen within the first week of TB treatment. The standard regimen is specified by contemporary TB treatment guidelines^6,8^. In brief, a prescription of isoniazid, rifampin or rifabutin, ethambutol, or pyrazinamide was considered standard treatment at the commencement of the intensive phase of TB treatment (within 7 days). The definition of treatment interruption was that all TB medications had been stopped for more than one day.

### Group classification

Patients without initial use of the standard regimen were identified and classified as the non-standard TB (NSTB) group. From the patients with standard initial TB treatment, we selected a control group (STB group) in a ratio of 1:1 who were matched by age (within a difference of 5 years), sex, and year of initial TB treatment.

### Clinical characteristics and laboratory data

We retrieved the participant’s clinical information such as age, sex, body weight, body mass index, smoking status and underlying co-morbidities from the hospital’s electronic record database. Co-morbidities including eye diseases, diabetes mellitus (DM), chronic obstructive pulmonary disease (COPD), other chronic lung disease, liver disease (hepatitis B or C virus infection, cirrhosis of liver, and liver cancer), gout or hyperuricemia, chronic kidney disease (CKD), long-term dialysis, autoimmune disease, active cancer, transplantation, and prior tuberculosis history were coded by the Nurse Practitioners (Chen RT and Liu CY), who reviewed medical records and used a standard case report form with default options. CKD was defined as at least stage 3^9^. Current smoker was defined as those who had smoked > 100 cigarettes with the last time of smoking within one month before entering the study^10^. Laboratory data were collected, including grade of acid-fast smear, hemoglobin, liver transaminases, total bilirubin and blood uric acid. Hyperuricemia was defined as a blood uric acid level greater than 7.0 mg/dL.

We recorded the possible clinical reasons for not initially prescribing the standard anti-TB regimen. In addition, the treatment duration and the occurrence of anti-TB treatment interruption, which was defined as stopping all anti-TB medications, were coded as long-term outcomes.

### Statistical analysis

Analysis between the NSTB and STB groups was performed. Categorical variables were analyzed by chi-squared test and continuous parameters by Student’s *t*-test. Logistic regression was used for risk factors associated with the NSTB group. The crude odds ratios (OR) were obtained by univariate logistic regression. If variables had p < 0.10, they were entered into the multi-variable logistic regression analysis by forward conditional stepwise methods. Statistical significance was set to p < 0.05. The statistical analyses were performed in SPSS version 19 (IBM, Chicago, IL, USA).

### Ethics approval and consent to participate

The Research Ethics Committee of National Taiwan University Hospital approved this study (IRB No.: 202209085RIND). Written informed consent was waived by the Research Ethics Committee of the study hospital due to the retrospective study design.

## Results

### Patient recruitment

**Figure 1:**
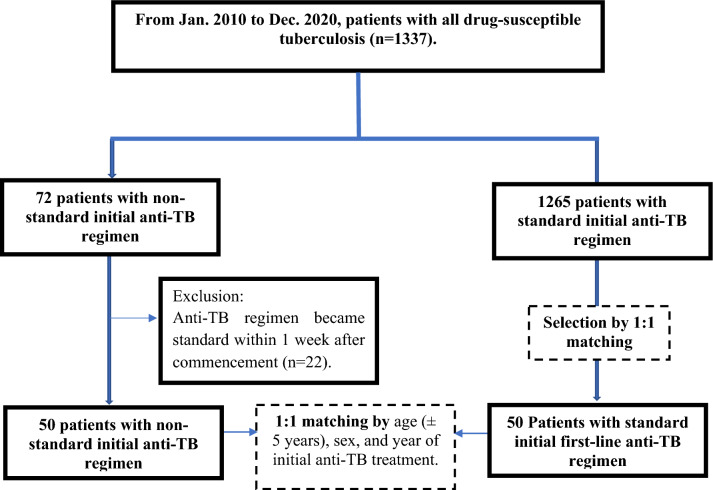
Flow chart of participant enrollment. *TB* tuberculosis.

From January 2010 to December 2020, a total of 1337 patients at the National Taiwan University Hospital were diagnosed with pulmonary tuberculosis and were also susceptible to all first-line anti-TB medications. Of them, 72 patients had non-standard initial anti-TB regimens but 22 patients resumed all four first-line standard anti-TB regimens within 1 week after TB treatment commencement. We finally analyzed the 50 (3.7%) patients with non-standard prescriptions at initial TB treatment (NSTB group) and selected 50 controls with standard initial anti-TB treatment for the drug susceptible TB (STB group) with 1:1 matching (Fig. 1). In this study, patients who started standard treatment and whose medication was changed due to side effects were not excluded from the study. We had recorded the patients whose medication was changed due to side effects 48 patients in STB group and more than NSTB group had 29 patients (96% vs. 58%, p < 0.005).

### Patient demographics

The clinical characteristics of the participants with pulmonary TB are listed in Table 1. Among them, there were six cases with combined pulmonary and extrapulmonary tuberculosis, including one of tuberculous spondylitis in the NSTB group and four of tuberculosis of pleura in the STB group. The 50 patients in the NSTB group and those in the STB group were similar in age, proportion of male gender, smoking status, prior TB history and body mass index. As for underlying co-morbid conditions, the proportions of the patients with eye disease (38% vs. 10%; p = 0.001), liver disease (53% vs. 12%, p < 0.001), hepatitis B or C infection (30% vs. 12%; p = 0.027), liver cirrhosis (10% vs. 0%; p = 0.022), and gout/hyperuricemia (36% vs. 14%; p = 0.011) were significantly different between the two groups. Another comorbidity in the NSTB group but not in the STB group was hepatocellular carcinoma (1% vs. 0%, p = 0.315). A total of six patients had hepatic failure. The Child Pugh classifications were class A in four patients and class B in one patient. All of them had cirrhosis before TB treatment. On the other hand, the initial sputum acid fast smear (AFS) grade and hemoglobin level were higher in the STB group (p = 0.038 and 0.006, respectively) than in the NSTB group, whereas AST and total bilirubin were significantly lower in the STB group (p = 0.044 and 0.047, respectively).

**Table 1:** Clinical characteristics of patients with standard or non-standard initial anti-tuberculosis treatment regimen.

Characteristics	Not standard initial TB treatment (N = 50)	Standard initial TB treatment (N = 50)	*p* value
Age: year	71.8 ± 15.3	71.7 ± 14.9	0.989
Male gender	39 (78%)	39 (78%)	1.000
Smoking			0.899
Current smoker	4 (8%)	5 (10%)	
Ex-smoker	11 (22%)	12 (24%)	
BW	56.0 ± 16.6	58.2 ± 14.1	0.472
BMI	21.6 ± 4.2	22.2 ± 3.3	0.484
Underlying co-morbid condition			
Eye disease	19 (38%)	5 (10%)	0.001
DM	14 (28%)	10 (20%)	0.349
COPD	10 (21%)	8 (16%)	0.603
Chronic lung disease (emphysema excluded)	6 (12%)	6 (12%)	1.000
Liver disease	26 (53%)	6 (12%)	< 0.001
Hepatitis B and C	15 (30%)	6 (12%)	0.027
Liver cirrhosis	5 (10%)	0(0%)	0.022
Hepatic carcinoma	1 (2%)	0 (0%)	0.315
Gout/hyperuricemia	18 (36%)	7 (14%)	0.011
ESRD	4 (8%)	0 (0%)	0.041
CKD	8 (16%)	3 (6%)	0.110
Cancer	16 (32%)	8 (16%)	0.061
Remission	13 (26%)	8 (16%)	0.220
Active	3 (6%)	1 (2%)	0.307
Autoimmune disease	1 (2%)	0	0.315
Post transplantation	2 (4%)	0	0.153
Prior TB history	3(6%)	2(4%)	0.646
Laboratory tests			
Initial acid-fast smear			0.038
Strong: 3+ to 4+	2 (4%)	10 (20%)	
Weak: 1+ to 2+	9 (18%)	10 (20%)	
Hemoglobin (g/dL)	11.1 ± 2.3	12.4 ± 2.4	0.006
AST (U/L)	43.3 ± 41.5	30.3 ± 16.9	0.044
ALT (U/L)	40.4 ± 57.0	24.3 ± 24.0	0.070
Total bilirubin (mg/dL)	1.8 ± 3.6	0.7 ± 0.5	0.047
Treatment duration, days	281 ± 121	223 ± 63	0.003
Treatment interruption	13 (26%)	4 (8%)	0.021

Data are no. (%) or mean ± standard deviation and analyzed by Chi-squared and Student’s *t*-test, respectively.

*ALT* alanine aminotransferase, *AST* aspartate aminotransferase, *BMI* body mass index, *BW* body weight, *COPD* chronic obstruction pulmonary disease, *DM* diabetes mellitus, *TB* tuberculosis.

For the treatment course, the NSTB group had longer treatment durations (281 ± 121 vs. 223 ± 63 days; p = 0.003) and more occurrences of treatment interruption (26% vs. 8%; p = 0.021) than the STB group did. The anti-TB medication was changed due to side effects in 22 patients in the STB group and 23 in the NSTB group (44% vs. 43%, p = 0884). The 4 patients lost to follow up were in the NSTB group, with none in the STB group (8% vs. 0%; p = 0.041), whereas the 1 death was in the STB group (0% vs. 1%, p = 0.315).

### The drugs not prescribed in the initial anti-TB regimen

The medications that were not prescribed to patients who received the non-standard initial anti-TB treatment are listed in Table 2. Pyrazinamide (60%) was the drug that was most commonly not prescribed in the initial NSTB group, followed by ethambutol (34%). The other two were rifampin (16%) and isoniazid (8%), which were not excluded as often. The clinical reasons for not prescribing the intact standard anti-TB regimen at initial treatment were commonly liver disease (53%) and eye disease (38%). Underlying eye disease with poor visual acuity was found in 19 patients, including color vision dysfunction in 4 patients, macular lesion in 1 patient, cataract in 8 patients, amblyopia in 1 patient, glaucoma in 2 patients, and blurred vision without definite cause in 3 patients. The eye disease in the enrolled patients existed before tuberculosis treatment. Among the 26 patients in the NSTB group with liver disease, there were 3 patients with liver cirrhosis and 2 with liver cirrhosis and hepatocellular carcinoma (four Child Pugh class A and one class B). In addition, there were thirteen patients with chronic viral hepatitis B or C and one with hepatocellular carcinoma. Of these, two had received a liver transplantation. All of them had liver disease before TB treatment. Others reasons included gout/hyperuricemia (36%), frailty (old age and multiple comorbidities) (18%), skin rash (4%), and unclassified causes (12%).

**Table 2:** Medications and clinical reasoning for patients with non-standard initial anti-tuberculosis treatment.

The drug not prescribed in initial anti-TB regimen^a^	
Isoniazid	4 (8%)
Rifampin	8 (16%)
Ethambutol	17 (34%)
Pyrazinamide	30 (60%)
The clinical reasons for exclusion of drugs in standard initial anti-TB regimen^b^	
Eye disease	19 (38%)
Skin rashes	2 (4%)
Elderly age or multiple comorbidities	9 (18%)
Liver disease	26 (53%)
Gout/hyperuricemia	19 (38%)
Other	4 (8%)

^a^Five patients did not receive > 1 first-line anti-TB drugs. Four did not receive isoniazid, rifampin, or pyrazinamide. One did not receive rifampicin or pyrazinamide.

^b^There were 2 patients with 4 reasons, 5 with 3 reasons, and 13 with 2 reasons for not being prescribed the standard initial anti-TB regimen.

*TB* tuberculosis.

### Logistic regression for risks associated with non-standard initial anti-TB regimen

We conducted a logistic regression analysis for risk of NSTB at initial anti-TB treatment (Table 3). The significant pre-treatment factors (p < 0.010) determined by the univariate analysis were entered into the multivariate logistic regression model using stepwise methods. Eye disease (adjust odds ratio [aOR]: 8.869; 95% CI 2.542–30.949; p = 0.001), gout/hyperuricemia (aOR: 4.012; 95% CI 1.196–13.425; p = 0.024), and liver disease (aOR: 12.790; 95% CI 3.981–41.089; p < 0.001) were shown to be independent factors in the final logistic regression model.

**Table 3:** Logistic regression for non-standard initial TB treatment.

Factors	Multivariable	
	Adjusted HR (95% CI)	*p* value
Eye disease	8.869 (2.542–30.949)	0.001
Liver disease	12.790 (3.981–41.089)	< 0.001
Gout/hyperuricemia	4.012 (1.196–13.425)	0.024

Significant pre-treatment factors (p < 0.010) in univariate analysis are entered in the multivariable regression.

The factors in the final model were selected by the forward conditional stepwise methods.

*TB* tuberculosis.

## Discussion

In the present study, non-standard initial anti-TB regimens were prescribed to 3.7% of the first-line drug susceptible pulmonary TB patients. Pyrazinamide and then ethambutol were the first-line anti-TB drugs that were most commonly avoided at the initiation of treatment. The independent risk factors for non-standard initial TB treatment were underlying liver disease, eye disease and hyperuricemia/gout. In the NSTB group, the patients had significantly longer anti-TB treatment durations and more anti-TB treatment interruptions.

Actually, the use of the standard anti-TB treatment regimen at the initiation of therapy is important to decrease the bacterial burden and improve the outcome^11^. A previous study reported that the adherence rate to anti-TB drug doses was not 100% and suggested several strategies to improve it^12^. On the other hand, no studies have investigated the prescription of non-standard anti-TB regimens at initial treatment, which is also a clinical concern. In our study, 5.3% of patients with drug-susceptible TB were prescribed non-standard regimens, and 1.5% of them began receiving the standard regimen within 7 days. The remaining 3.7% (n = 50) were studied, and notably, this population was associated with prolonged treatment course and treatment interruption. Although no causal relationship could be proven in this case control study, the association with negative impacts requires attention and further investigation.

The main clinical reasons for initially prescribing a nonstandard anti-TB regimen were underlying liver disease and eye disease. Underlying liver disease is prevalent in the study area, which has a relatively high viral hepatitis infection rate^13,14^, and is one of risk factors for drug related liver injury during TB treatment^15,16^. Before clinicians prescribe an anti-TB regimen, they may be concerned about abnormal liver transaminase or bilirubin levels. Pyrazinamide was the drug most commonly excluded in the non-standard regimen, possibly due to its high hepatotoxicity^17^ and anti-TB role for active TB bacilli compared with isoniazid and rifampicin^18^. In fact, research has shown that standard treatment, including PZA, is effective and safe for patients with hepatitis C or liver cirrhosis^19,20^. These findings suggest that treatment for TB in HCV-seropositive patients could be pursued in the usual manner, using standard short-course regimens, with the condition that monthly liver function tests must be performed.

In addition, hyperuricemia and gout were independent factors for a non-standard anti-TB regimen, especially for pyrazinamide, which may induce these kinds of side effects^21^. However, hyperuricemia is not a contraindication for using PZA. Without pyrazinamide, however, the duration will be prolonged and the extent of TB bacilli eradication will be a concern. Clinical efforts to prescribe the standard anti-TB regimen are recommended whenever possible.

The second most common reason for non-standard anti-TB regimen was underlying eye disease, which is a concern when prescribing ethambutol^22^. Because of the irreversibility of some ethambutol-related ocular side effects, including optic neuritis, clinicians might check the baseline examination before prescribing this drug, especially to those with poor visual acuity, underlying eye disease or complaints. Although monthly monitoring by an ophthalmologist is recommended, ocular side effects are reportedly not detected by regular checks of visual acuity and color vision^23^. For patients at high risk of ocular toxicity, ethambutol might be still prescribed after initial examinations by an ophthalmologist. However, close monitoring with point-of-care methods is recommended over regular examinations.

There are several limitations in this study. First, this study was a retrospective case control design. The examinations and treatments were not standardized, and no causal relationships could be confirmed. In addition, we recorded the causes of TB medication discontinuation by chart review, and most of the reasons were based on clinical judgement, warranting further investigation. Third, the case number was small, and a further large-scale study is warranted. Fourth, this study was conducted in Taiwan, so generalization of the findings to other areas or ethnicities might require validation.

In conclusion, non-standard anti-TB regimens were prescribed at initial treatment to 3.7% of the patients with drug-susceptible TB. The main clinical reasons were underlying liver disease, eye disease and hyperuricemia/gout, which might raise concerns about drug side effects during TB treatment. However, a nonstandard anti-TB regimen was associated with prolonged treatment course and treatment interruption. Subsequent studies to improve the nonstandard anti-TB regimen in the intensive phase are needed.

## Data Availability

The datasets used and/or analysed during the current study available from the corresponding author on reasonable request.
